# Boosting Photoresponse Performance and Stability of Photoelectrochemical Photodetectors by Chemical Bath Depositing Multilayer MoS_2_ on ZnO Electrode

**DOI:** 10.3390/nano15120875

**Published:** 2025-06-06

**Authors:** Jingyao Ma, Jiawei Wang, Xin Shi, Tianqi Sun, Pengpeng Dai

**Affiliations:** Xinjiang Key Laboratory of Luminescence Minerals and Optical Functional Materials, School of Physics and Electronic Engineering, Xinjiang Normal University, Urumqi 830054, China

**Keywords:** photoelectrochemical photodetector, ZnO/MoS_2_, chemical bath deposition, stability, rapid response

## Abstract

ZnO nanorods are promising nanomaterials for photoelectrochemical photodetectors (PEC PDs). However, the weak photocurrent density, delayed response, and low stability of ZnO are major drawbacks for their applications. To address these challenges, we integrated multilayer MoS_2_ nanosheets with ZnO nanorods using a chemical bath deposition method. The resulting ZnO/MoS_2_ heterojunction achieved a photocurrent density of 1.02 mA/cm^2^ (~20 times higher than that of bare ZnO), ultrafast response times (90/150 ms), and 92% stability retention over 3600 s. These enhancements originated from suppressed charge recombination and accelerated water oxidation kinetics. Our work provides another possible energy-saving route toward developing high-efficiency and stable ZnO-based photoanodes for practical applications in PEC PDs.

## 1. Introduction

Photoelectrochemical (PEC) photodetectors (PDs) convert light into electrical signals using a photoelectrochemical process. They have made rapid strides in the fields of information devices and energy conversion owing to their low cost, simple production method, low working voltage, and high sensitivity. In recent years, PD devices based on zinc oxide (ZnO) nanostructures have found extensive application because of their direct bandgap semiconductor properties, strong electron mobility, great chemical stability, high exciton binding energy (60 meV), eco-friendly nature, and cost-effectiveness [1,2,3]. In particular, aligned ZnO nanorod arrays can absorb and trap more incident photons, which lowers the channel resistance and improves the device’s conductivity. Moreover, the directional transport of photogenerated carriers through the vertical structure can efficiently enhance the electron–hole pair separation [4]. Nevertheless, the poor photocurrent density, slow response (often > 1000 ms), and low stability of pure ZnO are significant disadvantages for its PD applications [5]. Constructing a heterostructure using ZnO nanorods and suitable semiconductor materials is a practical way to break the above restrictions.

MoS_2_ is an ideal candidate material for mitigating the aforementioned limitations of ZnO due to its superior optoelectronic properties, including a high internal quantum efficiency (~85%); an exceptional on/off current ratio (~10^7^), surpassing that of graphene-based devices by several orders of magnitude (*I_light_*/*I_dark_* ≈ 4 in the literature) [6]; and good carrier mobility (>200 cm^2^ V^−1^ s^−1^) [7]. In addition, MoS_2_ and ZnO can easily form a type-II heterojunction, which is conducive to improving the photocurrent density and speeding up the response time by increasing the spacing of photogenerated carriers. For example, Lamouchi et al. constructed a ZnO/MoS_2_ heterostructure to observe the benefits of a type-II heterojunction: its photocurrent density value was ~7 times higher than that of pure ZnO [8]. Gautam et al. created ZnO/MoS_2_ PDs, which had a faster response time (*t_res_* = 2.46 s) than pure ZnO PDs (*t_res_* = 3.75 s) [9]. Ma et al. studied ZnO/MoS_2_ PDs, which also exhibited fast response/recovery times (*t_res_*/*t_rec_* = 0.15 s/0.17 s) [10]. In addition, the stability of ZnO-based electrodes, primarily limited by the oxidation kinetics of photogenerated holes at the electrode/electrolyte interface, can be enhanced through a rational heterostructure design that leverages the abundant active sites at MoS_2_ edges to accelerate photoelectrochemical reaction kinetics [11]. Different deposition methods, such as chemical vapor deposition, solgel-assisted synthesis, dual-phase solid-state dewetting, and drop casting, can be used to deposit MoS_2_ on a ZnO surface for photodetector applications [9,10,12,13]. Nevertheless, there are still desirable, cheaper, and efficient high-performance techniques with a lower thermal budget (i.e., the total thermal energy input required during fabrication [14]) and without a special gas environment or high-power demand for creating controlled heterostructures based on MoS_2_.

Chemical bath deposition (CBD) is a versatile technique frequently used in solar cells [15], sodium-ion batteries [16], gas sensors [17], and supercapacitors [18] due to its low processing temperature (typically below 100 °C), reproducibility, easy-to-change parameters, and low cost. Additionally, this technique enables the optimization of films’ thickness, grain size, crystalline quality, and shape by regulating a growth parameter, such as deposition time, molarity, solution temperature, and so on. Considering these advantages, employing CBD to deposit MoS_2_ on ZnO could be a successful strategy with which to obtain superior quality ZnO/MoS_2_ PEC PDs.

To improve the main factors restricting the application of ZnO PEC PDs, such as weak photocurrent density, delayed response, and low stability, an energy-efficient CBD method was used in this study to prepare ZnO/MoS_2_ heterojunctional PEC PDs. The microstructure of the ZnO/MoS_2_ photoanode and the layer number information of MoS_2_ in the heterojunction were analyzed via transmission electron microscopy (TEM) and Raman spectroscopy, and photoelectrochemical activity, PEC PD performance, and stability were investigated in detail. EIS spectra, Bode phase plots, and open-circuit photovoltage vs. time curve measurements suggest that the incorporation of MoS_2_ could improve the interface electron transfer, resulting in an enhanced PEC PD property. Based on the results, a possible mechanism is proposed to explain the enhanced performance of ZnO/MoS_2_ photoanodes. The results offer a new insight into the development of efficient and stable heterogeneous PEC PDs.

## 2. Materials and Methods

### 2.1. Preparation of the ZnO Photoanode

Before the hydrothermal reaction, 5 mL of zinc acetate solution (5 mM, Macklin, Shanghai, China) was dropped onto an ITO conductive substrate. After drying, it was calcined at 350 °C for 2 h. Subsequently, the pre-treated conductive substrate was immersed in a reaction solution consisting of hexamethylenetetramine (25 mM, SCR, Shanghai, China) and zinc nitrate hexahydrate (25 mM, SCR, Shanghai, China) dissolved in 14 mL of water, followed by the hydrothermal reaction at 90 °C for 3 h. The hydrothermal growth process was repeated three times to obtain ~1.58 μm long and ~137 nm wide ZnO nanorods on ITO. Then, the ZnO photoanode was ultrasonicated in anhydrous ethanol for 5 min, followed by rinsing in deionized water for another 5 min under ultrasonication. This ethanol–water cleaning cycle was repeated three times to ensure the thorough removal of loosely adhered particles. Finally, the sample was dried at 60 °C for 6 h in a vacuum drying oven.

### 2.2. Fabrication of the ZnO/MoS_2_ Photoanode

**Figure 1:**
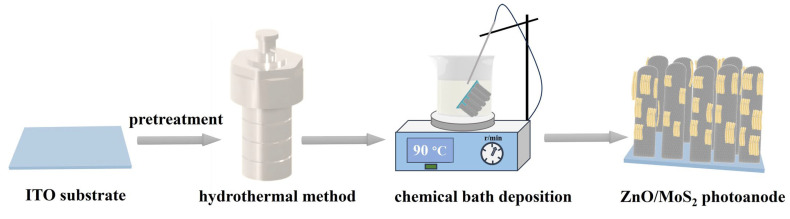
A schematic representation of the preparation procedure for the ZnO/MoS_2_ photoanode.

The ZnO/MoS_2_ photoanode was prepared using the CBD method. Firstly, (NH_4_)_2_MoS_4_ (26 mg, 99.95% purity, Alfa, NJ, USA) was dissolved in 50 mL of deionized water to achieve a 3.93 mM solution. Then, 100 μL of hydrazine hydrate (80% AR, Kermel, Tianjin City, China) was added to the above solution. After that, the ZnO photoanode (~1.58 μm length, ~137 nm width nanorods on the ITO surface) was positioned in the solution at a 45° angle to the beaker wall (ZnO-coated side pointing downward), and deposition proceeded at 90 °C and 200 rpm stirring for 40 min to fabricate the ZnO/MoS_2_ photoanode, with an extended reaction time to 80 min yielding the ZnO/MoS_2_-80 heterojunction for comparison. The preparation procedure of the ZnO/MoS_2_ photoanode is illustrated in Figure 1.

### 2.3. Characterization

TEM (FEI Tecnai G2 F20, FEI, Hillsboro, OR, USA) was used to evaluate the samples’ morphologies and microstructures. Prior to TEM testing, we scraped the nanorods off the ITO substrate using a diamond blade. The collected material was then subjected to ultrasonic dispersion in anhydrous isopropyl alcohol at 40 kHz for 10 min to isolate individual nanorods for TEM characterization. The Raman spectra of the as-fabricated materials were obtained using a Horiba Jobin Yvon LabRam apparatus (Paris, France.) via excitation with a 532 nm laser.

### 2.4. Photoelectrochemical Measurements

A three-electrode (as-prepared electrode, Pt wire, and Ag/AgCl electrode) electrochemical workstation (Chenhua, Shanghai, China.) configuration was implemented to test the photoelectrochemical properties of the produced photoanodes. The light source was a xenon lamp (100 mW/cm^2^). The electrolyte was an aqueous solution comprising a combination of 0.1 M Na_2_S·9H_2_O and 0.02 M Na_2_SO_3_. Cyclic linear sweep voltammetry was used to measure the I–V curves from −1 to 1 V vs. Ag/AgCl at a scan rate of 30 mV/s. The transient photocurrent response was measured at the potential of 0.1 V vs. Ag/AgCl with a switching duration of 5 s. Under open-circuit voltage conditions, the EIS spectra were recorded in the frequency range of 0.01 Hz to 100 kHz. A diagram of the ZnO/MoS_2_ PEC PDs performance test is illustrated in Figure S1 in Supporting Information.

## 3. Results and Discussion

**Figure 2:**
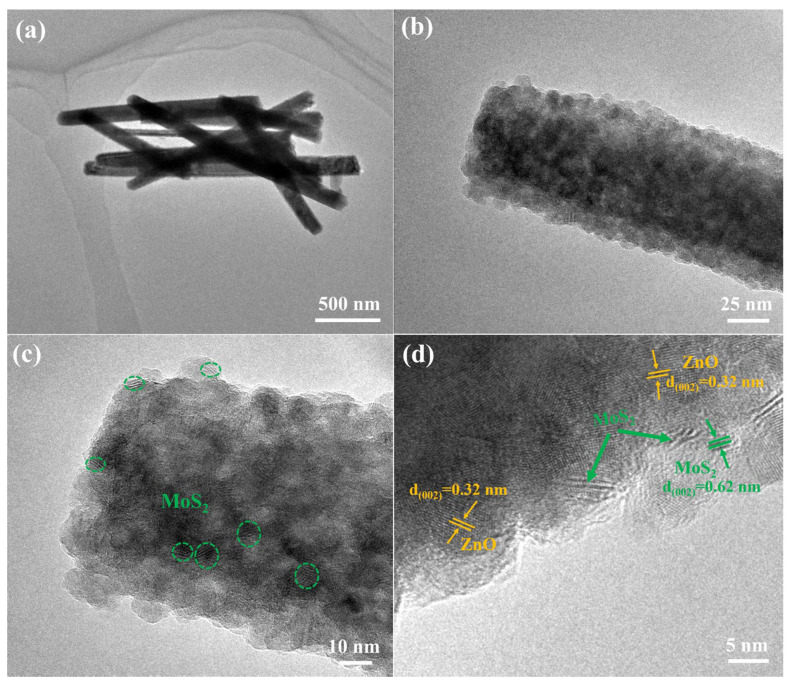
(**a**) TEM images of ZnO/MoS_2_ heterostructure, (**b**,**c**) progressively enlarged TEM photographs of ZnO/MoS_2_ heterostructure, the part surrounded by the green dotted circle is MoS_2_. (**d**) high-resolution TEM imaging of ZnO/MoS_2_ heterostructure.

The morphologies and microstructure of the produced ZnO/MoS_2_ heterostructure were investigated via transmission electron microscopy (TEM) and high-resolution TEM (HRTEM). As seen in Figure 2a, the ZnO/MoS_2_ heterojunction had a uniform nanorod-like appearance, with the average length and width being approximately 1.58 μm and 137 nm, respectively. The TEM images, after magnification (Figure 2b), show that the ZnO nanorods were composed of small particles, while MoS_2_ was not observed. The further enlarged TEM images (Figure 2c) show that the ZnO surfaces were adorned with several MoS_2_ nanosheets, and the lattice spacing could be determined based on the clear view provided by the HRTEM images (Figure 2d). A lattice spacing of 0.62 nm was indexed to the (002) plane of 2H-MoS_2_ [19]. Additionally, a 0.32 nm lattice spacing appeared in good agreement with the (0110) plane of ZnO [20]. The TEM characterization revealed the successful formation of a ZnO/MoS_2_ heterostructure, which would facilitate the transfer of photogenerated carriers.

**Figure 3:**
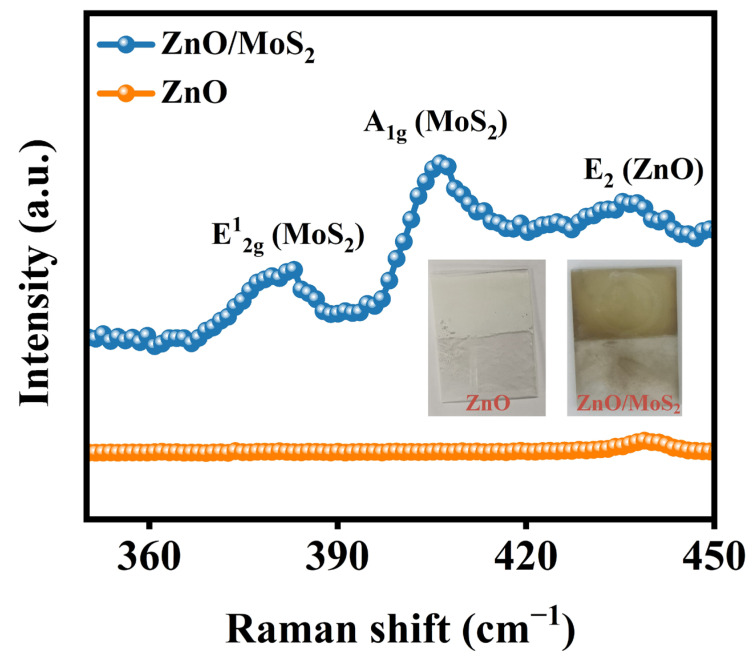
Raman spectra of ZnO and ZnO/MoS_2_ photoanodes.

Raman spectra were further obtained to investigate the chemical structure of the ZnO/MoS_2_ photoanode. As illustrated in Figure 3, the pure ZnO photoanode exhibited a peak situated at roughly 438 cm^−1^, which was indexed to the *E_2_* mode of ZnO [21]. Conversely, the ZnO/MoS_2_ photoanode exhibited two obvious peaks at 381 cm^−1^ and 406 cm^−1^, in addition to the ZnO characteristic peaks, corresponding to the in-plane lattice vibration ($E_{2g}^{1}$) and out-of-plane lattice vibration ($A_{1g}$) of MoS_2_ [22]. The peak value difference between $E_{2g}^{1}$ and $A_{1g}$ was 25 cm^−1^, indicating that MoS_2_ in the ZnO/MoS_2_ photoanode was four-layered (multilayer; ≥3 layers) [23,24]. This value aligns with the reported values for a pure MoS_2_ photodetector, while multilayer MoS_2_ demonstrated enhanced light absorption capacity compared to few-layer structures in optoelectronic applications [25]. Moreover, a leftward shift in the ZnO peak position was observed in the heterojunction compared to pristine ZnO, which was attributed to strong interfacial electronic interactions between the two semiconductors [26,27]. Figure S2 displays the ZnO/MoS_2_-80 Raman spectrum. We can see that the photoanode has two distinct distinctive peaks that belong to MoS_2_ at 380 cm^−1^ and 406 cm^−1^, with a peak separation of 26 cm^−1^, exceeding the 25 cm^−1^ value in ZnO/MoS_2_-40, indicating that the thickness of MoS_2_ increased with the increase in reaction time [28]. However, the peak of E_2_-ZnO is invisible, potentially due to laser shielding by the MoS_2_ shell. Prior studies on ZnO-based nanocables have also noted similar occurrences [29]. Figure S3 shows the statistical results for the Raman spectra data for multiple positions of the ZnO/MoS_2_ photoanodes. The average peak positions of MoS_2_ in the ZnO/MoS_2_ heterojunction were 380 ± 0.81 cm^−1^ ($E_{2g}^{1}$ peak of MoS_2_) and 405.6 ± 0.63 cm^−1^ ($A_{1g}$ peak of MoS_2_). The small deviations indicated the uniform deposition of MoS_2_ on the ZnO surface.

**Figure 4:**
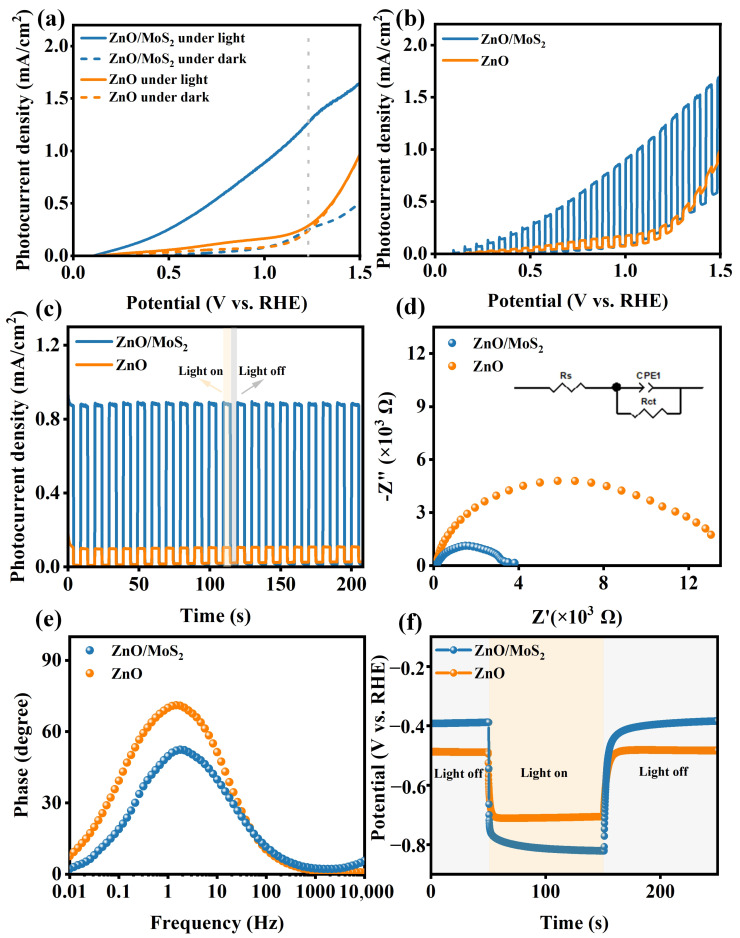
The PEC properties of the ZnO and ZnO/MoS_2_ photoanodes: linear sweep curves (**a**) with and without illumination, (**b**) with chopped illumination, (**c**) I-t curves, (**d**) EIS curves (the inset is the equivalent circuit model), (**e**) Bode phase plots, and (**f**) open-circuit photovoltage vs. time curves.

Linear sweep curves were determined for the pure ZnO and ZnO/MoS_2_ heterojunction photoanodes using a typical three-electrode configuration under continuous and chopped 100 mW/cm^2^ illumination (Figure 4a,b). The ZnO/MoS_2_-40 photoanode showed noticeably higher photocurrent densities throughout the entire potential window compared to its ZnO and ZnO/MoS_2_-80 counterparts, achieving 1.02 mA/cm^2^ at 1.23 V vs. RHE (the standard reversible potential for water oxidation), which was ~20 times and ~4.6 times greater than that of the pure ZnO (0.05 mA/cm^2^) and ZnO/MoS_2_-80 (0.22 mA/cm^2^, Figure S4a,b) photoanodes, respectively. Additionally, the initial potentials of the ZnO, ZnO/MoS_2_-40, and ZnO/MoS_2_-80 photoanodes were approximately 136 mV, 110 mV, and 127 mV, respectively. The ZnO/MoS_2_-40 photoanode exhibited a significantly lowest initial potential value, indicating that optimal MoS_2_ modification effectively reduce the water oxidation energy barrier of ZnO, thereby promoting the water oxidation kinetics [28]. The above results determined that ZnO/MoS_2_-40 was the structure with the highest light response intensity.

The photocurrent responses of the ZnO and ZnO/MoS_2_ photoanodes at 1 V vs. RHE under multiple switching cycles are also shown in Figure 4c. The ZnO/MoS_2_ photoanode had a greater photocurrent response intensity than the pure ZnO photoanode, demonstrating that its electronic lifetime and separation efficiency are significantly higher than those of a single ZnO photoanode. Furthermore, with instantaneous light-switching intervals, we could see that every manufactured photoelectrode displayed a consistent increase and decrease in photocurrent density, indicating their high photo-responsiveness and reversibility. Even after 200 s of rapid switching, the ZnO/MoS_2_ photoanode’s photocurrent density and reproducibility remained constant, demonstrating its great stability and consistency.

Impedance spectra of the ZnO and ZnO/MoS_2_ photoanodes were obtained to study the dynamics of photogenerated charge transport, as shown in Figure 4d. The corresponding equivalent circuit model is presented in the inset, where *Rₛ*, *CPE*, and *R_ct_* represent the series resistance between the photoanode and the ITO substrate, the constant phase element, and the charge transfer resistance at the photoanode–electrolyte interface, respectively. The measured *R_s_* values for the ZnO and ZnO/MoS_2_ photoanodes were 120.0 Ω and 121.9 Ω, respectively. The minimal difference in the *R_s_* values indicates that their impedance spectra were recorded under similar environments, enabling a direct comparison of the *R_ct_* values. Specifically, the *R_ct_* values were as follows: ZnO/MoS_2_ (3.14 kΩ) < ZnO (12.32 kΩ). The significantly lower *R_ct_* value of the ZnO/MoS_2_ photoanode suggests that interfacial engineering effectively enhances the charge separation efficiency while accelerating the surface water oxidation kinetics [30]. This result supports the photocurrent response performance observed in Figure 4c.

The Bode diagram of the ZnO and ZnO/MoS_2_ photoanodes more directly reflects the influence of heterojunction construction on the carrier dynamics (Figure 4e). According to previous reports, the photoanodes displayed greater phase values at low-frequency peaks, suggesting that the charge transfer at the electrode/electrolyte interface significantly restricted the oxidation of the electrode water [31,32]. As shown in Figure 4e, the characteristic peaks of the ZnO/MoS_2_ and ZnO photoanodes are both at low frequencies (around 1 Hz), and the low-frequency peak of ZnO/MoS_2_ photoanode is significantly lower than that of the ZnO photoanode, revealing quicker charge transfer kinetics at the electrode/electrolyte interface of the ZnO/MoS_2_ photoanode.

The open-circuit photovoltage Δ*OCP* (*V_light_* − *V_dark_*) curves of the as-fabricated photoanodes were determined to identify the band bending degree and the surface charge transfer mechanism. As illustrated in Figure 4f, the Δ*OCP* value of the ZnO/MoS_2_ photoanode was 0.42 V, which is ~52.4% higher than that of pristine ZnO (0.22 V). This suggests that the ZnO/MoS_2_ photoanode has quicker charge transfer and greater band bending at its photoanode/electrolyte interface.

**Figure 5:**
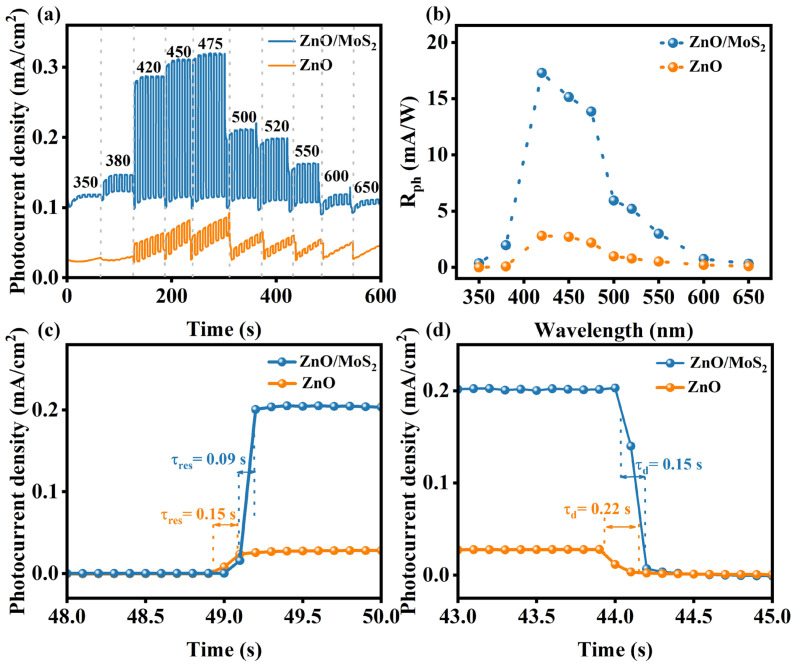
The performances of the ZnO and ZnO/MoS_2_ PEC PDs: (**a**) response curves under various wavelengths, (**b**) corresponding *R_ph_* values, (**c**) *t_res_* values at 475 nm light irradiation, and (**d**) *t_rec_* values at 475 nm light irradiation.

The sensitivity performance of the ZnO/MoS_2_ and ZnO PEC-type PDs at different indicative light wavelengths (350, 380, 420, 450, 475, 500, 520, 550, 600, and 650 nm) was investigated. As displayed in Figure 5a, compared with the ZnO PEC PD, the photoresponse of the ZnO/MoS_2_ PEC PD was more significant for each single-channel light, which can be ascribed to the efficient charge separation via type-II heterojunction formation between ZnO and MoS_2_. In addition, the *I_ph_* values of the ZnO/MoS_2_ PEC PD increased at a wavelength from 350 to 475 nm, and a maximum *I_ph_* value of ~210 μA/cm^2^ at 475 nm was achieved, which is seven times greater than that of the ZnO PEC PD (~30 μA/cm^2^) under the same conditions. As the wavelength redshifted from 500 to 650 nm, the *I_ph_* of the ZnO/MoS_2_ PEC PD decreased. Through power law analysis [33], we obtained that the weak capture coefficients of ZnO and ZnO/MoS_2_ PDs were 1.21 and 1.14, respectively (as shown in Figure S5, Supporting Information. The lower γ for ZnO/MoS_2_ indicates a reduction in the capture effect due to interface charge transfer. Moreover, the rejection ratio (RR = *I_ph_* (@475 nm, ~210 μA/cm^2^)/*I_ph_* (@350 nm, ~3 μA/cm^2^)) for the fabricated heterojunction device was ~70, which indicates that the photodetector exhibits a relatively high signal-to-noise ratio. The corresponding photoresponsivity (*R_ph_*) values (*R_ph_ = I_ph_/P*) of the ZnO/MoS_2_ and ZnO PEC-type PDs at different wavelengths were also calculated (Figure 5b). The ZnO/MoS_2_ photoanode exhibited a superior photoresponse (*R_ph_)* within the 350–600 nm range compared to the ZnO nanorods, which might be driven by dual enhancement mechanisms. Firstly, as shown in Figure S7, the structurally ordered ZnO nanorods can absorb and utilize light in the ultraviolet–visible range, which is due to their highly organized structure inducing significant light-scattering effects [10]. These scattering effects synergize with the intrinsic visible-light absorption capacity of multilayer MoS_2_, collectively improving the spectral utilization across the specified range. Furthermore, the type-II heterojunction at the ZnO/MoS_2_ interface facilitates a directional carrier transport while suppressing recombination losses, as validated by charge dynamics analyses in prior heterostructure studies [34,35]. The strategic coupling of these spectral broadening structural features with optimized interfacial charge transfer pathways constitutes the fundamental origin of the enhanced *R_ph_* performance. When the wavelength was extended from 350 to 420 nm, the associated *R_ph_* increased from 0.36 to 17.29 mA/W before progressively falling to 0.32 mA/W for the ZnO/MoS_2_ PEC PD. At 420 nm light irradiation, the highest *R_ph_* value (17.29 mA/W) of the ZnO/MoS_2_ PEC PD was recorded and appeared to be almost 6.2 times higher than that of the ZnO PEC PD (2.8 mA/W). The ZnO/MoS_2_ photoanode showed the highest response in the visible region, which can be attributed to the synergistic effect of light scattering by the ZnO nanorods and intrinsic light absorption in the electrolyte [36,37]. Additionally, it was also much more significant than that reported for pure MoS_2_ PDs (0.17 mA/W), also outperforming the ZnO/MoS_2_ PDs constructed using the drop-casting and chemical vapor deposition methods [10,13,38]. Thus, ZnO/MoS_2_ PDs constructed via chemical bath deposition have great potential for visible photodetection applications. Furthermore, the achieved responsivity exceeded that of ZnO heterojunctions modified with other 2D materials such as WS_2_ (2.42 mA/W) and graphene (17.1 mA/W) [39,40]. Also, the NEP and D of ZnO/MoS_2_ were calculated to evaluate the performance of the photodetector [6]. As shown in Figure S8a,b, the NEP and D were found to be 9.4 × 10^−12^ WHz^−1/2^ and 1.2 × 10^11^ Jones at a wavelength of 420 nm, respectively. Furthermore, we measured the EQE of the ZnO/MoS_2_ heterojunction PEC photodetector, as presented in Figure S8c. The EQE increased from 0.15% to 5.12% (350 nm–420 nm) before progressively falling to 0.08% (650 nm), which corresponds to the photocurrent and photoresponsivity dependence on the incident light wavelength. The highest EQE was 5.12% under illumination at 420 nm.

One important metric to precisely assess the photodetection sensitivity of PDs is the response/recovery time (*t_res_*/*t_rec_*). *t_res_* is the time needed to increase the maximum photocurrent value from 10% to 90%, and *t_rec_* is that needed to decrease it from 90% to 10% [5]. Figure 5c,d show the *t_res_* and *t_rec_* values of the ZnO/MoS_2_ and ZnO PDs under 475 nm light irradiation. We can observe that *t_res_* and *t_rec_* for the ZnO/MoS_2_ PD were 90 ms and 150 ms, respectively, but for the ZnO PD, they were 150 ms and 220 ms, respectively. Both the *t_res_* and *t_rec_* for the ZnO/MoS_2_ PD were remarkable smaller than those for the ZnO PD, which suggests that heterojunction construction can lessen the recombination between electrons and holes in the photodetector, enhancing the device’s optoelectronic performance. Furthermore, the achieved response/recovery times outperformed those of ZnO heterojunctions modified with other two-dimensional layered materials, such as ZnO/rGO (200/200 ms) and graphite/ZnO–WS_2_ (14.27/52.63 s), demonstrating the superior charge transport properties of MoS_2_-modified architectures in photodetection applications [41,42].

**Figure 6:**
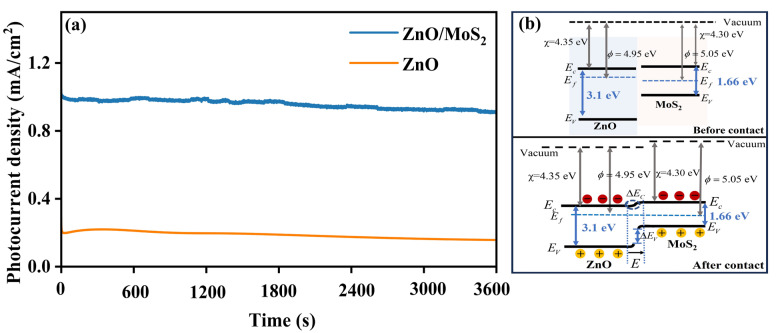
(**a**) Stability test of ZnO/MoS_2_ and ZnO PEC PDs, (**b**) schematic ZnO/MoS_2_ heterojunction band diagram (*Φ*: work function; *χ*: electron affinity; *E_f_*: Fermi level; *E_c_*: conduction band bottom; *E_v_*: valence band top; *E*: built-in electric field).

Another essential characteristic for the practical use of PEC PDs is their high stability. Therefore, a preliminary photoelectrochemical stability screening of the ZnO/MoS_2_ and ZnO PDs was carried out. As depicted in Figure 6a, following 3600 s of testing, the pure ZnO PD underwent photocorrosion, reaching a photocurrent density reserve of only about 76% of their original photocurrent density. By comparison, the ZnO/MoS_2_ PDs sustained around 92% their initial photocurrent density, demonstrating exceptional PEC stability and also superiority to ZnO-based photoanodes previously reported (e.g., Al-ZnO/CdS [43], ZnO:Co@ZIF-8 [44]). The mechanism behind the enhanced photoresponse of the ZnO/MoS_2_ heterostructure lies in the staggered energy levels between ZnO (3.1 eV) and four-layer MoS_2_ (1.66 eV). As shown in Figure 6b, the differences in work function (4.95 eV vs. 5.05 eV) and electron affinity (4.35 eV vs. 4.30 eV) between ZnO and MoS_2_ induce electron diffusion from ZnO to MoS_2_ upon contact, in order to balance the Fermi levels [34,45]. Thus, the heterostructure’s energy band diagram experiences a minimum bending effect, greatly expanding the range of states that charge carriers can reach. The device’s photoresponse is greatly increased by this increased concentration in charge carriers, which increases its efficiency in transforming photonic energy into an electrical response [33]. Concurrently, the low charge transfer resistance at the heterojunction/electrolyte interface and the numerous active sites in multilayer MoS_2_ synergistically accelerate hole depletion via the Na_2_S/Na_2_SO_3_ sacrificial agent, thus minimizing hole accumulation and associated oxidative corrosion. Table 1 lists the characteristic properties of typical ZnO/MoS_2_ PDs. In contrast to ZnO/MoS_2_ PDs formed using other techniques, our proposed ZnO/MoS_2_ PEC PDs formed using the CBD method showed a quick response time. The stability of photodetectors can be evaluated through different curves. Our work and [10] recorded the changes in the detector’s output signals (such as photocurrent or voltage) over time under constant light and bias conditions. Refs. [25,46] describe the changing trends during the long-term monitoring of performance parameters (*R_ph_*) under accelerated aging conditions such as high temperature, high humidity, and continuous light exposure. We can observe that our proposed ZnO/MoS_2_ PEC PD has comparable long-term stability. The lower responsivity (*R_ph_*) values of our PEC-type photodetector compared to those of its traditional counterparts in Table 1 arise from energy losses inherent to the light-to-chemical conversion for driving the redox reactions, whereas conventional detectors directly transduce light into electrical signals without such energy dissipation [46].

## 4. Conclusions

In this work, we adopted an energy-saving CBD method to deposit molybdenum disulfide nanosheets (~4 layers) on zinc oxide nanorods for photoelectrochemical photodetectors, which were shown to possess fast response times (*t_res_* = 90 ms/*t_rec_* = 150 ms) and peak responsivity of 17.29 mA/W under illumination wavelength of 420 nm, along with an outstanding D (EQE) value of 1.2 × 10^11^ Jones (5.12%) and an excellent noise-equivalent power of 9.4 × 10^−12^ WHz^−1/2^. Further, the ZnO/MoS_2_ PEC PDs exhibited long-term stability, retaining 92% of their initial photocurrent density throughout 3600 s of testing. This work provides an effective strategy for developing low-energy-consumption and highly stable MoS_2_-based-heterojunction visible-light photodetectors, Furthermore, the low-cost and low-temperature processing characteristics of this method establish a technical foundation for the scalable fabrication of large-area, self-powered optoelectronic devices based on low-dimensional semiconductor materials in the future.

## Figures and Tables

**Table 1:** Performance comparison between previously published ZnO/MoS_2_ PEC PDs manufactured by depositing MoS_2_ nanosheets on ZnO using various techniques and PEC PDs based on ZnO/MoS_2_, manufactured using the CBD method.

Materials	Synthesis Method of Heterojunction	*R_ph_* (mA/W)	*t_res_* (s)/*t_re_*_c_ (s)	Stability	Refs.
MoS_2_	Electrophoretic deposition method	0.17	0.3/0.3	/	[38]
ZnO-MoS_2_	Solgel-assisted synthesis method	34.50	2.46/4.82	/	[9]
ZnO/MoS_2_/HNP	Dual-phase solid-state dewetting approach	1430	3.37/0.35	/	[12]
ZnO/MoS_2_	Drop-casting method	751	7/29.1	/	[13]
nf-MoS_2_/Si_3_N_4_	Radio frequency (RF) sputtering	1358	/	1170 mAW^−1^ at 100 °C	[25]
MoS_2_	Mechanically exfoliated	140–138,400	68.6 × 10^−6^/100 × 10^−6^	80% retained	[47]
ZnO/monolayer MoS_2_	Chemical vapor deposition methods	4	0.15/0.17	21,600 s~98% retained	[10]
ZnO/MoS_2_	Chemical bath deposition method	17.29	0.09/0.15	3600 s~92% retained	This work

## Data Availability

The data that support the plots within this paper and other findings of this study are available from the corresponding author upon reasonable request.

## Supplementary Materials

The following supporting information can be downloaded at: https://www.mdpi.com/article/10.3390/nano15120875/s1, Figure S1: Diagram of the ZnO/MoS_2_ PEC PDs performance test; Figure S2: (a) Raman spectra of different points on the ZnO/MoS₂ photoanode; (b) the statistical results of Raman spectra data; Figure S3: ABPE curves of ZnO and ZnO/MoS_2_ photoanodes; Figure S4: Raman spectra of ZnO/MoS_2_-80 photoanodes; Figure S5: Power law-fitted graph for various wavelengths of illumination; Figure S6: The PEC properties of ZnO/MoS_2_-80 photoanodes: Linear sweep curves (a) with and without illumination (b) with chopped illumination, (c) EIS curves, with the corresponding Bode phase plots shown in the inset, (d) Open circuit photovoltage vs. time curves; Figure S7: UV–vis absorption spectra of ZnO photoanodes, with the corresponding Tauc plots shown in the inset; Figure S8: (a) NEP, (b) D and EQE for the ZnO/MoS_2_ heterostructure-based device.
