# Supplementary material for: Boosting Photoresponse Performance and Stability of Photoelectrochemical Photodetectors by Chemical Bath Depositing Multilayer MoS2 on ZnO Electrode
Source: Nanomaterials (Basel). 2025 Jun 6;15(12):875. doi: 10.3390/nano15120875 (PMC12196496; doi:10.3390/nano15120875)
Supplement: Supplementary file 1 [file nanomaterials-15-00875-s001.zip › nanomaterials-3672747-supplementary.pdf]

nanomaterials

Article

Boosting photoresponse performance and stability of photoelectrochemical photodetectors by chemical bath depositing multilayer MoS_2 on ZnO electrode

Jingyao Ma*, Jiawei Wang, Xin Shi, Tianqi Sun, Pengpeng Dai*

Xinjiang Key Laboratory for Luminescence Minerals and Optical Functional Materials, School of Physics and Electronic Engineering, Xinjiang Normal University, Urumqi, Xinjiang 830054, China
*Corresponding author: E-mail: majingyao@xjnu.edu.cn (J M), E-mail: daipp614@xjnu.edu.cn (P D).

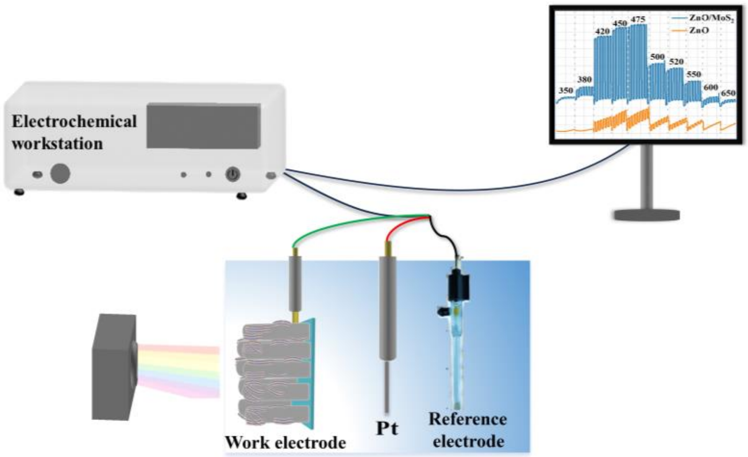

Figure S1. Diagram of the ZnO/MoS_2 PEC PDs performance test.

Fig. S2 shows the statistical results of Raman spectra data for multiple positions of ZnO/MoS_2 photoanodes, the average peak positions of MoS_2 in ZnO/MoS_2 heterojunction were 380 ±0.81 cm^{-1} (E^1_{2g} peak of MoS_2), 405.6±0.63 cm^{-1} (A_{1g} peak of MoS_2). The small deviations indicate the uniform deposition of MoS_2 on the ZnO surface.

Academic Editor: Firstname Lastname

Received: date
Revised: date
Accepted: date
Published: date

Citation: To be added by editorial staff during production.

Copyright: © 2025 by the authors. Submitted for possible open access publication under the terms and conditions of the Creative Commons Attribution (CC BY) license (https://creativecommons.org/licenses/by/4.0/).

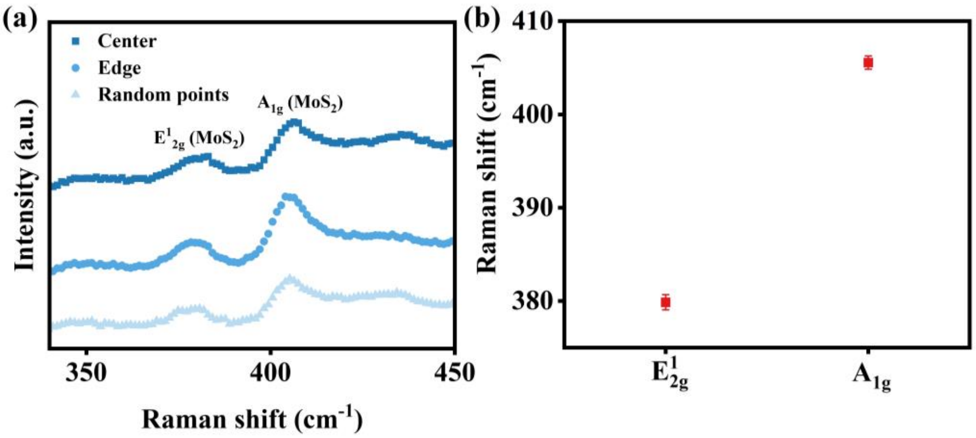

Figure S2. (a) Raman spectra of different points on the ZnO/MoS_2 photoanode; (b) the statistical results of Raman spectra data.

The applied bias photon-to-current efficiency (ABPE) of our fabricated electrodes is shown in Fig. S3. The ZnO/MoS_2 heterostructure achieved a maximum ABPE of 0.25% at 0.78 V vs. RHE, representing an 8.3-fold enhancement compared to pristine ZnO (0.03% at 0.83 V vs. RHE) while operating at a 50 mV lower potential. The remarkable improvement in photoconversion efficiency under reduced bias voltage highlights the synergistic advantages of the heterojunction architecture, particularly its enhanced charge separation and reduced overpotential requirements, which are critical for enabling efficient self-biased operation in practical photoelectrochemical (PEC) devices.

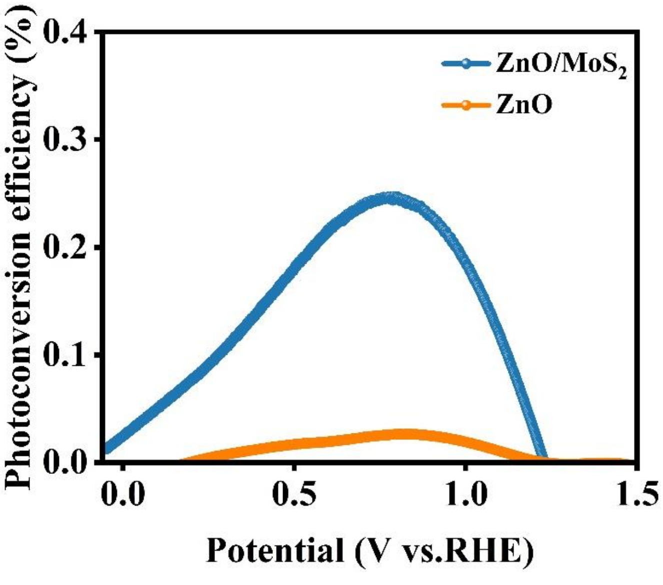

Figure S3. ABPE curves of ZnO and ZnO/MoS_2 photoanodes.

Figure S4 displays the ZnO/MoS_2-80 Raman spectrum. We can see that the photoanode has two distinct distinctive peaks that belong to MoS_2 at 380 cm^{-1} and 406 cm^{-1}. However, the peak of E_2-ZnO is invisible, potentially due to laser shielding by the MoS_2 shell. Prior studies on ZnO-based nanocables have also noted similar occurrences[29].

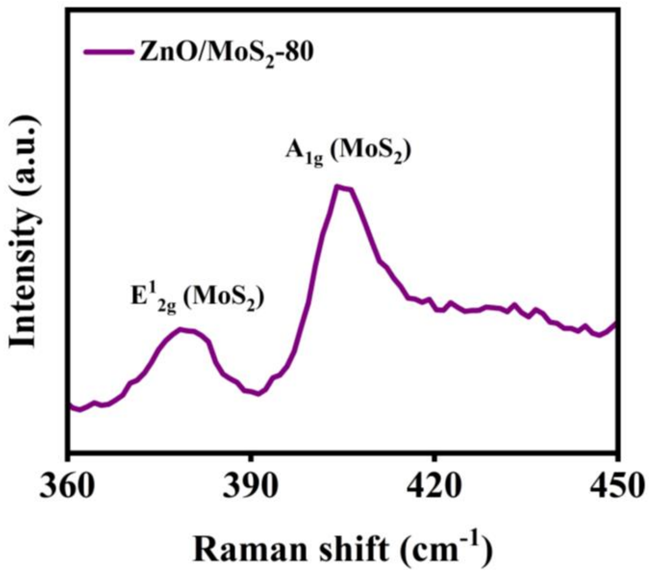

Figure S4. Raman spectra of ZnO/MoS_2-80 photoanodes.

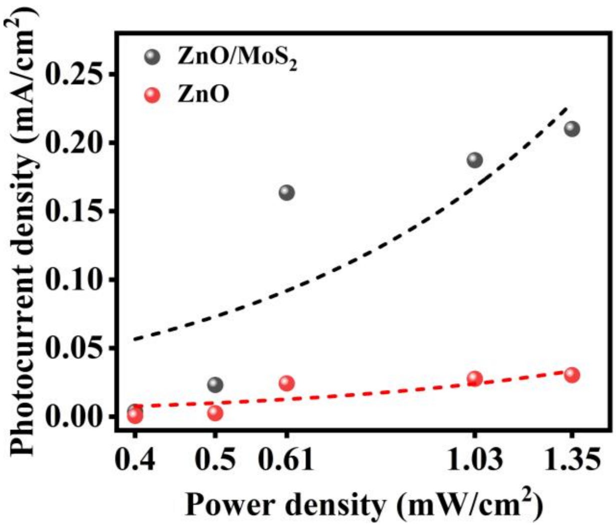

Figure S5. Power law-fitted graph for various wavelengths of illumination.

The PEC properties of ZnO/MoS_2-80 heterojunction photoanode was studied: The Linear sweep curves under continuous and chopping 100 mW/cm^2 illumination (Fig. S6a and b) show that the initial potential of the ZnO/MoS_2-80 photoanode is approximately 127 mV. Under the bias voltage of 1.23 V vs. RHE, the photocurrent density reached 0.22 mA/cm^2. The impedance of the ZnO/MoS_2-80 photoanode was further obtained as 6.52 Ω through electrochemical impedance spectroscopy. The bode phase diagram revealed that in the low-frequency region, the phase value of ZnO/MoS_2-80 was between ZnO and ZnO/MoS_2-40. The V-t curves reveals that its open-circuit voltage is 0.33 V. Compared with the pure ZnO, the ZnO/MoS_2-80 exhibits enhanced photoelectrochemical properties, which are attributed to the heterojunction effect. However, its performance is lower than that of ZnO/MoS_2-40. This is because excessive MoS_2 deposition on the surface of ZnO will increase the carrier migration distance, thereby increasing the carrier recombination rate

and weakening the performance. The above results indicate that ZnO/MoS_2-40 has the best performance.

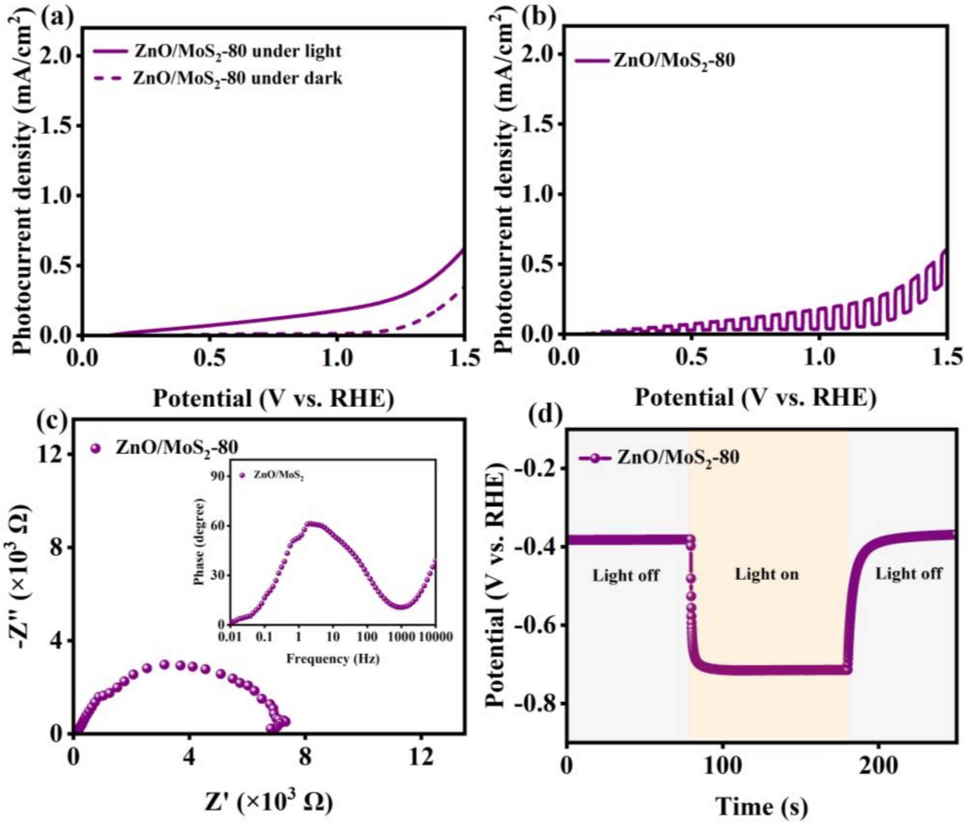

Figure S6. The PEC properties of ZnO/MoS_2-80 photoanodes: Linear sweep curves (a) with and without illumination (b) with chopped illumination, (c) EIS curves, with the corresponding Bode phase plots shown in the inset, (d) Open circuit photovoltage vs. time curves.

The optical absorption property of the ZnO was tested, and the measured absorbance spectrum and the corresponding Tauc plots shown is shown in Fig. S7. The ZnO photoanode demonstrated pronounced UV absorption characteristics due to its intrinsic bandgap energy (~3.1 eV). Significantly, the well-aligned nanorod architecture induced pronounced light scattering phenomena, which synergistically enhanced optical absorption across the visible spectrum through extended photon propagation paths[10].

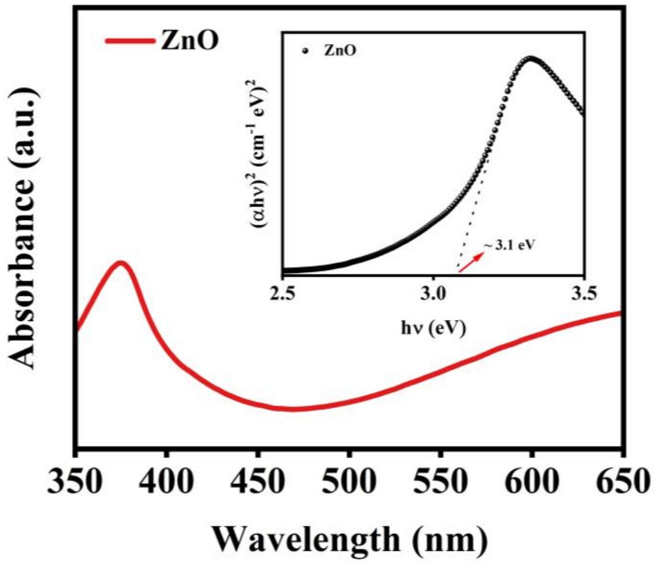

Figure S7. UV–vis absorption spectra of ZnO photoanodes, with the corresponding Tauc plots shown in the inset.

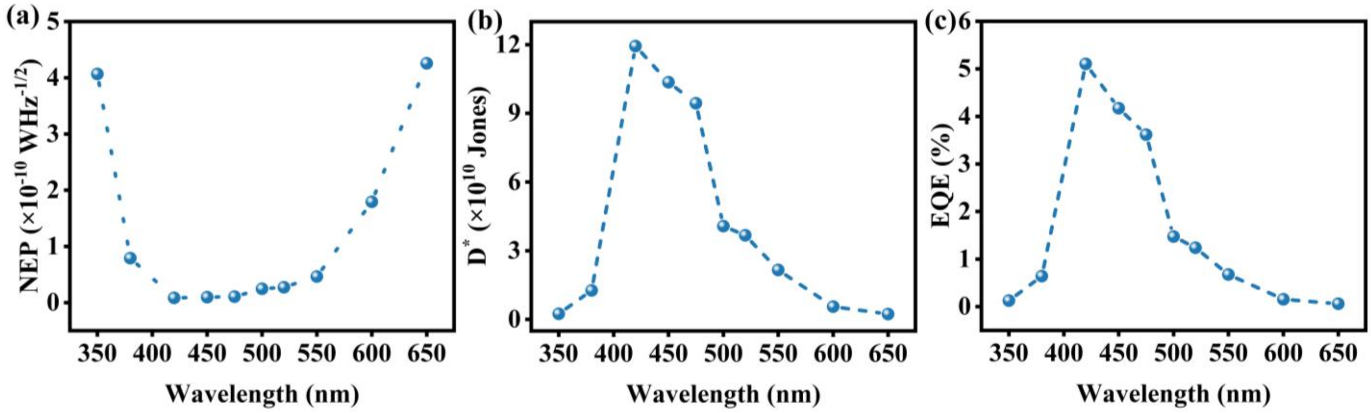

Figure S8. (a) NEP, (b) D and EQE for the ZnO/MoS_2 heterostructure-based device.
